# Perfect Topological Metal CrB_2_: A One-Dimensional (1D) Nodal Line, a Zero-Dimensional (0D) Triply Degenerate Point, and a Large Linear Energy Range

**DOI:** 10.3390/ma13194321

**Published:** 2020-09-28

**Authors:** Yang Li, Jihong Xia, Rabah Khenata, Minquan Kuang

**Affiliations:** 1 Department of Physics, Chongqing University of Arts and Sciences, Chongqing 402160, China; 2 Laboratoire de Physique Quantique de la Matiere et de Modelisation Mathematique (LPQ3M), Universite de Mascara, Mascara 29000, Algeria; 3 School of Physical Science and Technology, Southwest University, Chongqing 400715, China

**Keywords:** DFT, CrB_2_ material, linear band crossings, topological metal, spin–orbit coupling, P6/mmm, electronic structures

## Abstract

Topological materials with band-crossing points exhibit interesting electronic characteristics and have special applications in electronic devices. However, to further facilitate the experimental detection of the signatures of these band crossings, topological materials with a large linear energy range around the band-crossing points need to be found, which is challenging. Here, via first-principle approaches, we report that the previously prepared P6/mmm-type CrB_2_ material is a topological metal with one pair of 1D band-crossing points, that is, nodal lines, in the *k_z_* *=* 0 plane, and one pair of 0D band-crossing points, that is, triple points, along the A–Γ–A’ paths. Remarkably, around these band-crossing points, a large linear energy range (larger than 1 eV) was found and the value was much larger than that found in previously studied materials with a similar linear crossing. The pair of nodal lines showed obvious surface states, which show promise for experimental detection. The effect of the spin–orbit coupling on the band-crossing points was examined and the gaps induced by spin–orbit coupling were found to be up to 69 meV. This material was shown to be phase stable in theory and was synthesized in experiments, and is therefore a potential material for use in investigating nodal lines and triple points.

## 1. Introduction

Topological semimetals/metals [1,2,3,4,5,6,7,8,9,10,11,12,13,14,15], including nodal point semimetals/metals, nodal line semimetals/metals, and nodal surface semimetals/metals, with nontrivial band topologies, have attracted significant attention in the area of condensed matter physics. Topological semimetals/metals can be roughly classified based on the dimensionality of the band crossings. In nodal point topological materials, the bands cross at isolated points. For example, in Weyl semimetals/metals [12], the valence band and conduction band touch at an isolated nodal point in the momentum space of solids. Around the isolated Weyl points, the low-energy quasiparticles are described using Weyl fermions with a definite chirality. If the system is protected from proper crystal symmetry, the opposite chirality of the two Weyl points can coincide at a certain point, resulting in a Dirac point. To date, the investigation of nodal point semimetals/metals has not been limited to the cases of Weyl and Dirac nodal point materials [16,17,18,19,20] with two-fold and four-fold degenerated band crossings. Some novel nodal point materials with three-fold, six-fold, and eight-fold degenerated band crossings have also been predicted. Significant efforts have been devoted to the prediction of nodal-point materials that can feature three-fold [21,22,23,24,25], six-fold [26,27], and eight-fold degenerated [28] band crossings.

In nodal line semimetals/metals [29,30,31,32,33,34,35,36,37,38] and nodal surface semimetals/metals [38,39,40,41], the band-crossing points form one-dimensional (1D) nodal lines and two-dimensional (2D) nodal surfaces, respectively, in the momentum space of solids. The nodal line materials were first predicted in carbon-based networks [42]. Nodal line materials have recently emerged as an interesting research topic due to their many interesting electronic and optical properties and their association with nontrivial drum-head-like surface states. Research on topological nodal surface materials is still in the preliminary stage, and many more in-depth physical problems still need to be solved [39].

Although some materials have been predicted to be nodal line or nodal point materials via first-principle calculations, the number of predicted triply degenerate nodal point semimetals/metals and the number of nodal line semimetals/metals is still limited. Importantly, for almost all of the previously studied topological materials, the linear energy range of the band dispersion around the band-crossing points is usually smaller than 1 eV [43]. It should be emphasized that this small linear energy range for the band dispersion adds a certain degree of difficulty to the subsequent experimental research.

In this work, by using first-principle approaches, we report a P6/mmm-type CrB_2_ topological metal with one pair of 1D band-crossing points, that is, 1D nodal lines, in the *k_z_* = 0 plane, and one pair of 0D band-crossing points, that is, triply degenerate points, along the A–Γ–A’ paths. CrB_2_, possessing a P6/mmm crystal structure, is an existing material [44], where the structural stability has been examined using phonon dispersion. Remarkably, a large linear band dispersion can be found around these band crossings, which should facilitate further experimental investigations. If spin–orbit coupling (SOC) is turned on, these band-crossing points become gapped; however, the gapped points have energies up to 69 meV. It should be noted that the gaps induced by spin–orbit coupling are smaller than other predicted topological materials with nodal line states. The CrB_2_ topological metal is a good candidate for investigating the physical properties of nodal point and nodal line fermions, as well as the relationship between them.

## 2. Methods and Materials

To calculate the electronic structure and the topological signature of the CrB_2_ material, first-principle approaches were realized by using the Vienna ab initio simulation package [45]. The generalized gradient approximation (GGA) of the Perdew–Burke–Ernzerhof (PBE) [46] function was adopted as the exchange-correlation potential. Moreover, the cutoff energy was set as 600 eV, and the Brillouin zone (BZ) was sampled using a Monkhorst–Pack *k*-mesh with a size of 12 × 12 × 11. To determine the nontrivial surface states in CrB_2_, the WANNIERTOOLS [47] package was used in this study.

**Figure 1:**
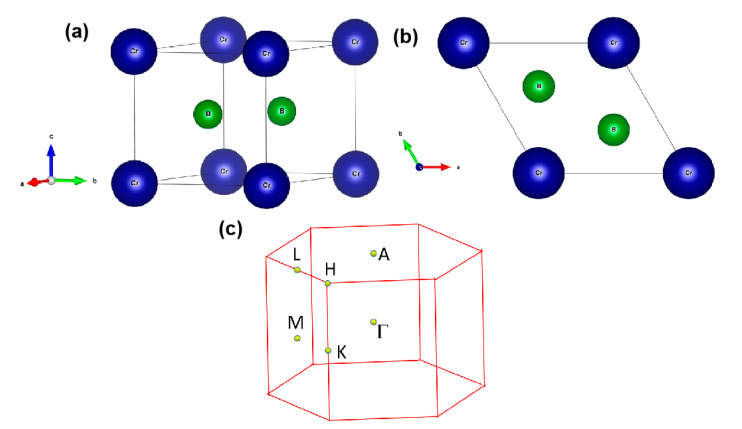
(**a**,**b**) Structural models of P6/mmm CrB_2_ from different viewpoints and (**c**) the bulk Brillouin zone of CrB_2_.

It should be highlighted that the CrB_2_ was prepared previously via direct synthesis from the elements using a method reported by Post et al. in 1954 [44]. The experimental lattice constants of CrB_2_ are reported to be a = b = 2.97 Å and c = 3.07 Å. The crystal structure of CrB_2_ was completely relaxed and the optimized lattice parameters are a = b = 2.97 Å and c = 2.93 Å. These values match well with those found in the experiment. The atomic positions are Cr at (0, 0, 0) and B at (0.66666, 0.333333, 0.5) and (0.333333, 0.66666, 0.5), and the corresponding relaxed crystal models under different views are shown in Figure 1a,b. Some information, including the elasticity, ICSD IDs, and the calculated X-ray diffraction can be found in the Materials Project database [48].

**Figure 2:**
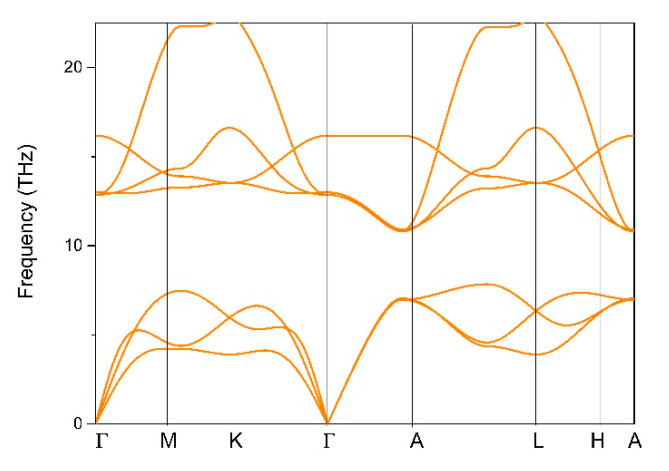
Phonon dispersion curve for CrB_2_.

Based on the crystal structure obtained for CrB_2_, the dynamical stability of CrB_2_ was examined based on the calculated phonon dispersion along the Γ–M–K–Γ–A–L–H–A paths (see Figure 1c). Usually, if the system does not contain imaginary frequencies in the phonon dispersion, it can be regarded as having dynamic stability [49,50,51]. As shown in Figure 2, the absence of imaginary frequencies in the first BZ reflects its dynamic stability.

## 3. Electronic Structures

**Figure 3:**
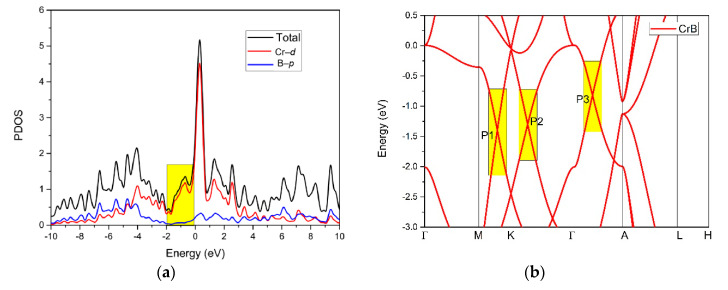
(**a**) The total and projected density of states (PDOS) of CrB_2_ without considering the spin–orbit coupling (SOC) effect and (**b**) the calculated band structure of CrB_2_ without SOC. The energy at the Fermi level was set to zero.

Figure 3a,b shows the calculated density of states (DOS) and the calculated band structure of the CrB_2_ material, respectively. As shown in Figure 3a, one can see an energy peak around the Fermi level, indicating that the CrB_2_ exhibited metallic properties. Moreover, based on the projected DOS, the total density of states in the energy range from −2 to 0 eV was mainly dominated by the Cr–*d* orbitals.

Figure 3b shows the calculated band structure of CrB_2_ along the Γ–M–K–Γ–A–L–H–A paths. From Figure 3b, we can see three obvious band-crossing points, named P1, P2, and P3, located along the M–K path, K–Γ path, and Γ–A path, respectively. For P1 and P2, they were two-fold degenerated nodal points; however, for P3, they were triply degenerate nodal points. Surprisingly, around these three nodal points, the energy range of the linear band dispersion was more than 1 eV (see the areas with a yellow background in Figure 3b). This large linear energy range was much larger than most of the other proposed materials with a linear-type band dispersion [43]. Moreover, this large linear energy range makes CrB_2_ a promising candidate for experimental studies on the interesting physics related to band crossings.

**Figure 4:**
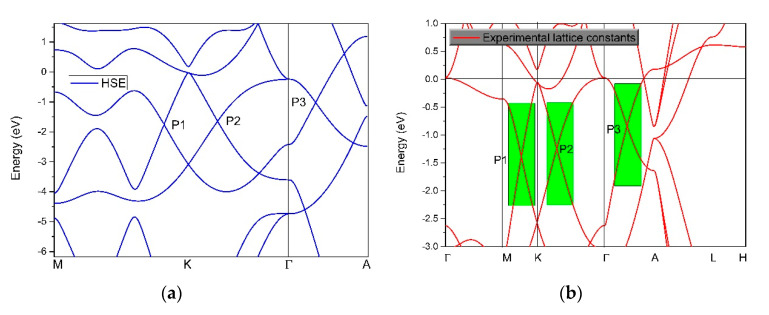
(**a**) The band structure of CrB_2_ calculated using the hybrid functional (HSE06) method and (**b**) the band structure of CrB_2_ using the generalized gradient approximation (GGA) method under experimental lattice constants.

Based on the hybrid functional (HSE-06) method [52], we further examined the band structure of CrB_2_ along the M–K–Γ–A paths. The HSE-06 method is usually used to obtain an accurate large range of the linear band dispersion and band-crossing points for topological materials. The results are given in Figure 4a, where one can see that all of the nodal points, that is, the two two-fold degenerated points and a triply degenerate point, were retained. In addition, the large energy range of the band dispersion was found close to these band-crossing points.

The band structure at the experimental lattice constants [44] was also calculated based on the GGA method, and the results are given in Figure 4b. By comparing Figure 3b and Figure 4b, we found that there was only a small difference between the two cases. In the linear band dispersion range, the energy bands of the two cases were basically consistent (see the areas with a yellow background in Figure 3b and the areas with a green background in Figure 4b).

## 4. Topological Signatures

In this section, we discuss the topological signatures of the three band-crossing points. The three band-crossing points can be classified into two types: (i) two-fold degenerated nodal points P1 and P2 and (ii) the triply degenerate nodal point P3. Based on the arguments made by Weng et al. [53], these doubly-degenerated crossings (i.e., P1 and P2) should belong in a line and these band-crossing points cannot be seen as isolated points. However, for the triply degenerate nodal point, this can occur both in isolation and at nodal line connections.

**Figure 5:**
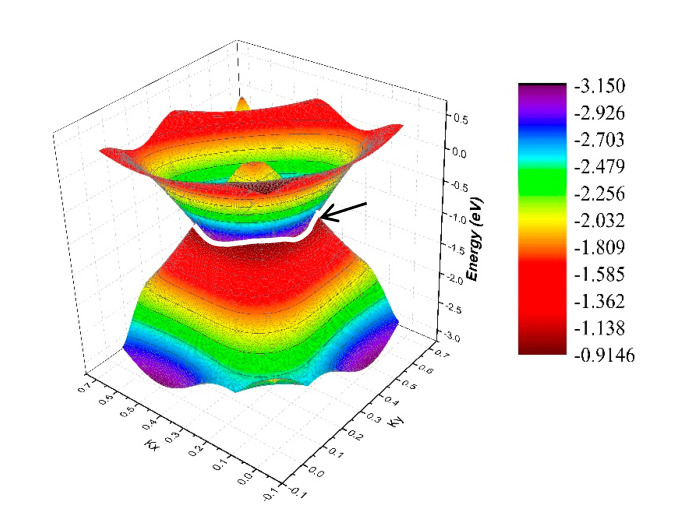
3D band dispersion around the K point in the *k_z_* = 0 plane. The white line shows the profile of the nodal line.

Let us first consider the two-fold band-crossing points P1 and P2. Based on the bulk BZ, as shown in Figure 1c, one can see that the P1 and P2 nodal points were all located in the *k_z_* = 0 plane. To prove that P1 and P2 reside on a nodal line, the K-centered three-dimensional (3D) plotting of the two bands in the *k_z_* = 0 plane is given in Figure 5. The white line in Figure 5 shows the intersections between the two bands, namely, an obviously closed line. As shown in Figure 5, we can see that the band-crossing points belonging to the nodal line are in the *k_z_ =* 0 plane, and this nodal line had a slight energy variation. More importantly, from this figure, we can conclude that there should be a series of two-fold degenerated nodal points, similar to P1 and P2, and around these points, there appeared to be a large energy range for the linear band crossings. It should be noted that this property should facilitate the experimental detection of the signatures of these band crossings. To capture the nature of the nodal lines, we calculated their Berry phase, as shown in Supplementary Materials Figure S1. From these calculations, we found that the Berry phase hosted a jump around the nodal line in the *k_z_* = 0 plane.

**Figure 6:**
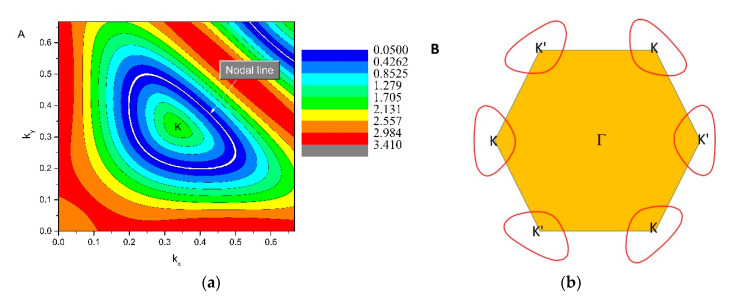
(**a**) 2D shape of the K-centered nodal line in the *k_z_* = 0 plane (highlighted by the white line) and (**b**) an illustration of one pair of nodal lines (highlighted by red lines) in the *k_z_* = 0 plane.

The 2D plane figure of the K-centered nodal line (located in the mirror-invariant *k_z_* = 0 plane) is shown in Figure 6a. For the CrB_2_ system, there were two independent mechanisms that protected the nodal line in the *k_z_* = 0 plane. The first was the mirror symmetry, as the CrB_2_ hosted a horizontal mirror plane; the other was the inversion symmetry and time-reversal symmetry. As the CrB_2_ hosted the time-reversal symmetry, one more nodal line could be found around the K’ point, as shown in Figure 6b.

**Figure 7:**
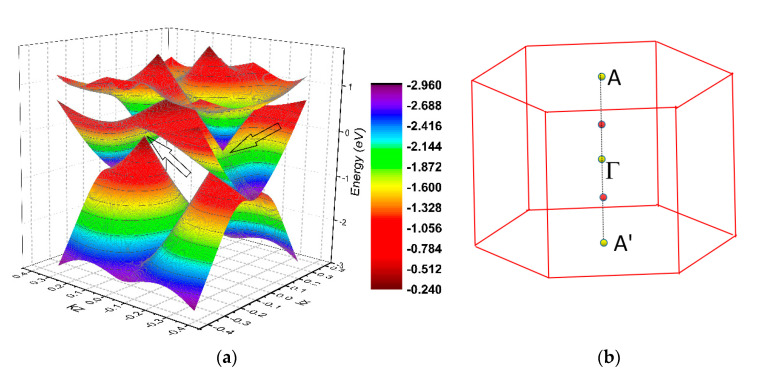
(**a**) 3D band dispersion along the A’–Γ–A paths in the *k_y_* = 0 plane. The arrows show the profile of the triply degenerate points and (**b**) the position of the triply degenerate points in the 3D Brillouin zone (BZ). The triply degenerate nodal points are highlighted using red balls.

We shall now discuss the triply degenerate nodal point, P3, along the Γ–A direction. These triply degenerate nodal points were formed by the crossing of a two-fold degenerated band and a non-degenerated band, which corresponded to the *E_1_* and *A_1_* irreducible representations, respectively, of the *C_6v_* symmetry. The 3D band dispersion along the A’–Γ–A paths in the *k_y_* = 0 plane is shown in Figure 7a, reflecting the occurrence of the triply degenerate nodal points. The arrows show the profile of the triply degenerate points. As the CrB_2_ system featured time-reversal symmetry, one pair of triply nodal points could be found, where one was located along the Γ–A path and the other was located along the Γ–A’ path. The position of the triply degenerate points in the 3D BZ is shown in Figure 7b.

Finally, it should be highlighted that the CrB_2_ system co-exhibited the 0D band crossing point, for example, the triply degenerate nodal points, and the 1D band-crossing points, that is, the nodal line states. Therefore, this material can be seen as having good potential prospects for studying the entanglement between the nodal point fermions and the nodal line fermions in the future.

## 5. Surface States and the Effect of Spin–Orbit Coupling

**Figure 8:**
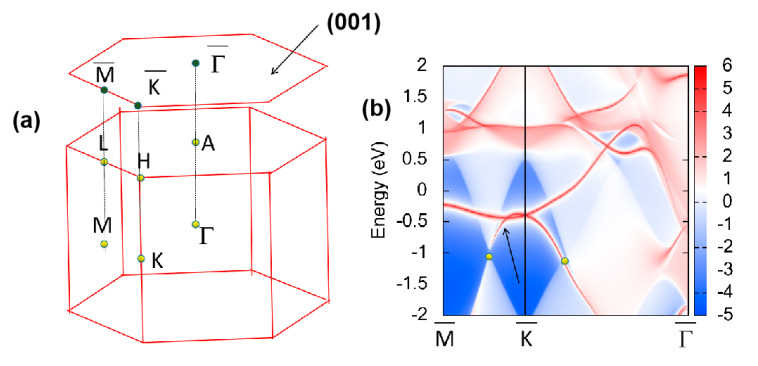
(**a**) 3D bulk BZ and 2D surface BZ and (**b**) projected spectrum on the (001) surface of CrB_2_. The band-crossing points and the nontrivial surface states are highlighted by the yellow balls and arrow, respectively.

Usually, 1D nodal lines enjoy nontrivial surface states. The K-centered nodal line in the *k_z_* = 0 plane was projected onto surface (001) (see Figure 8a), where the result is given in Figure 8b. From Figure 8b, we can observe that the nontrivial surface states (highlighted by the arrow) appeared around the band-crossing points (highlighted using yellow balls). These clear nontrivial surface states show promise for being detected in future experimental studies.

**Figure 9:**
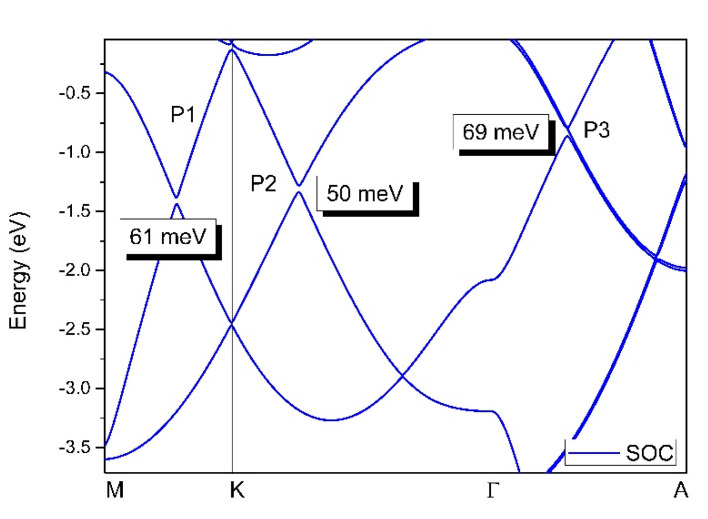
Band structure of CrB_2_ along the M–K–Γ paths calculated using a GGA. The spin–orbit coupling effect was considered.

Finally, we examined the effect of spin–orbit coupling on the electronic structure of the CrB_2_ system. The band structure of CrB_2_ investigated using the GGA method and the SOC effect are shown in Figure 9. It should be noted that almost all the previously predicted nodal line materials, triply degenerate nodal point materials, and SOC-induced gaps usually appear at these band-crossing points. For the CrB_2_ system, the SOC-induced gaps for the P1, P2, and P3 points were approximately 61, 50, and 69 meV, respectively. However, the spin–orbit coupling gaps in the CrB_2_ system were smaller than some of the other predicted topological materials, such as BaSn_2_ (>50 meV) [54], CaAgBi (>80 meV) [55], and TiOs (>100 meV) [56].

Finally, it should be further emphasized that topological materials with a 1D nodal line, 0D triply degenerate points, and a large energy range (>1 eV) for the linear band dispersion around band crossings have very rarely been reported in realistic materials [43]. Therefore, experimental confirmation of the novel properties proposed here is urgent.

## 6. Conclusions

Using first-principle approaches, we report that P6/mmm CrB_2_ was found to be a perfect topological metal with many interesting properties, where the conclusions of this study are summarized as follows:

1. CrB_2_ is an existing material and was confirmed to be dynamically stable based on the calculated phonon dispersion.
2. CrB_2_ featured two types of topological elements: (i) one pair of 1D nodal lines in the *k_z_* = 0 plane and (ii) one pair of 0D triply degenerate nodal points along the A’–Γ–A paths.
3. These band-crossing points were very robust against the effects of spin–orbit coupling.
4. The energy range of the linear band dispersion was very large around the band-crossing points.
5. The nontrivial surface states were around the band-crossing points and were very clear.
6. The large linear energy range, 0D and 1D band crossings, and obvious nontrivial surface states observed in CrB_2_ will facilitate the experimental detection of potential topological elements.

## Supplementary Materials

The following are available online at https://www.mdpi.com/1996-1944/13/19/4321/s1, Figure S1: The Berry phase along the M–K–Γ *k*-path for closed nodal line.
