# Supplementary material for: Perfect Topological Metal CrB2: A One-Dimensional (1D) Nodal Line, a Zero-Dimensional (0D) Triply Degenerate Point, and a Large Linear Energy Range
Source: Materials (Basel). 2020 Sep 28;13(19):4321. doi: 10.3390/ma13194321 (PMC7579166; doi:10.3390/ma13194321)
Supplement: Supplementary file 1 [file materials-13-04321-s001.pdf]

materials

Supplementary Materials

Perfect Topological Metal CrB_2: A One-Dimensional (1D) Nodal Line, a Zero-Dimensional (0D) Triply Degenerate Point, and a Large Linear Energy Range

1. Berry Phase Calculation

To capture the nature of the nodal lines, the Berry phase was computed, where the Berry phase was connected to the occupied Bloch band [1,2]. The result is shown in Figure S1. Obviously, the Berry phase experienced a jump around the nodal line in the $k_z = 0$ plane.

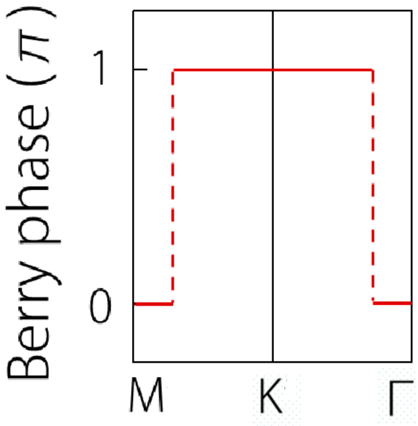

Figure S1. The Berry phase along the M–K–Γ *k*-path for closed nodal line.

References

1. Wan, X., Turner, A.M., Vishwanath, A., & Savrasov, S. Y. (2011). Topological semimetal and Fermi-arc surface states in the electronic structure of pyrochlore iridates. *Physical Review B*, 83(20), 205101.
2. Burkov, A. A., Hook, M. D., & Balents, L. (2011). Topological nodal semimetals. *Physical Review B*, 84(23), 235126.

© 2020 by the authors. Licensee MDPI, Basel, Switzerland. This article is an open access article distributed under the terms and conditions of the Creative Commons Attribution (CC BY) license (http://creativecommons.org/licenses/by/4.0/).
